# Impact of respiratory infectious epidemics on STEMI incidence and care

**DOI:** 10.1038/s41598-021-02480-z

**Published:** 2021-11-29

**Authors:** S. Macherey, M. M. Meertens, C. Adler, S. Braumann, S. Heyne, T. Tichelbäcker, F. S. Nießen, H. Christ, I. Ahrens, F. M. Baer, F. Eberhardt, M. Horlitz, A. Meissner, J. M. Sinning, S. Baldus, S. Lee

**Affiliations:** 1 Clinic III for Internal Medicine, University of Cologne, Faculty of Medicine and University Hospital Cologne, Kerpener Str. 62, 50937 Cologne, Germany; 2 Institute of Medical Statistics and Computational Biology, University of Cologne, Cologne, Germany; 3 Department of Cardiology, Augustinerinnen Hospital, Cologne, Germany; 4 Department of Cardiology, St. Antonius Hospital, Cologne, Germany; 5 Department of Cardiology, Evangelisches Krankenhaus Kalk, Cologne, Germany; 6 Department of Cardiology, Krankenhaus Porz am Rhein, Cologne, Germany; 7 Department of Cardiology, Krankenhaus Köln-Merheim, Cologne, Germany; 8 Department of Cardiology, St. Vinzenz Hospital, Cologne, Germany

**Keywords:** Cardiology, Interventional cardiology

## Abstract

The effect of respiratory infectious diseases on STEMI incidence, but also STEMI care is not well understood. The Influenza 2017/2018 epidemic and the COVID-19 pandemic were chosen as observational periods to investigate the effect of respiratory virus diseases on these outcomes in a metropolitan area with an established STEMI network. We analyzed data on incidence and care during the COVID-19 pandemic, Influenza 2017/2018 epidemic and corresponding seasonal control periods. Three comparisons were performed: (1) COVID-19 pandemic group versus pandemic control group, (2) COVID-19 pandemic group versus Influenza 2017/2018 epidemic group and (3) Influenza 2017/2018 epidemic group versus epidemic control group. We used Student’s t-test, Fisher’s exact test and Chi square test for statistical analysis. 1455 patients were eligible. The daily STEMI incidence was 1.49 during the COVID-19 pandemic, 1.40 for the pandemic season control period, 1.22 during the Influenza 2017/2018 epidemic and 1.28 during the epidemic season control group. Median symptom-to-contact time was 180 min during the COVID-19 pandemic. In the pandemic season control group it was 90 min (*p* = 0.183), and in the Influenza 2017/2018 cohort it was 90 min, too (*p* = 0.216). Interval in the epidemic control group was 79 min (*p* = 0.733). The COVID-19 group had a door-to-balloon time of 49 min, corresponding intervals were 39 min for the pandemic season group (*p* = 0.038), 37 min for the Influenza 2017/2018 group (*p* = 0.421), and 38 min for the epidemic season control group (*p* = 0.429). In-hospital mortality was 6.1% for the COVID-19 group, 5.9% for the Influenza 2017/2018 group (*p* = 1.0), 11% and 11.2% for the season control groups. The respiratory virus diseases neither resulted in an overall treatment delay, nor did they cause an increase in STEMI mortality or incidence. The registry analysis demonstrated a prolonged door-to-balloon time during the COVID-19 pandemic.

## Introduction

Multiple triggers of ST-elevation myocardial infarction (STEMI) were described previously, but the effect of respiratory infectious diseases on STEMI incidence is not well understood. The effect of these infectious diseases on treatment of patients with STEMI is not well investigated. The Influenza 2017/2018 season and the coronavirus disease 2019 (COVID-19) pandemic were chosen as observational periods to investigate the effect of respiratory virus diseases on STEMI incidence and treatment in a metropolitan area with a preexisting STEMI network. COVID-19 caused by severe acute respiratory syndrome coronavirus 2 (SARS-CoV-2) was declared a pandemic by the World Health Organization on March 11, 2020^1^. Previous studies and expert opinions around the world raised concerns about a trend towards decreased ST-elevation myocardial infarction (STEMI) admissions, pre-hospital treatment delay, as well as an overwhelmed medical system caused by the pandemic^2–7^. STEMI patients suffer from high mortality and morbidity rates, thus requiring prompt diagnosis and treatment^8^. After the initial outbreak in Wuhan, China in December 2019, the first case of COVID-19 was documented in Germany on January 27, 2020. On February 25, 2020, the first person in the German state of North Rhine Westphalia was diagnosed with COVID-19. The government of North Rhine Westphalia issued containment recommendations to hospitals on March 3, 2020, and passed a coronavirus executive order on March 22, 2020^9^. This lockdown resulted in contact restrictions, remote work and an extreme reduction of public life and mobility.

On the contrary, the Influenza 2017/2018 season—which was declared as epidemic in Europe and was a prototype of a seasonal respiratory infectious disease with high disease burden—did not lead to the implementation of comparable containment recommendations or executive orders^10^. The influenza season in general was described as predictor of increased incidence of myocardial infarction and might raise the cardiovascular risk of patients with coronary artery disease^11–13^. During the Influenza 2017/2018 epidemic in Germany 334,000 patients were diagnosed with influenza, of these, 60,000 required hospitalization^10^. The Influenza 2017/2018 epidemic resulted in a doubling of the registered influenza-caused deaths compared to the prior season 2016/2017^10^. The effect of this disease burden on the treatment of STEMI patients was not investigated before.

The current study aims to clarify the role of the recurrent influenza season on STEMI care exemplified by the 2017/2018 season. This study was also conducted to evaluate the hypothesized concerns about a decrease in STEMI admissions and treatment delay during the COVID-19 pandemic. Specifically, it addressed patient- and system-related delay in a myocardial infarction network in the city of Cologne, Germany^14,15^.

## Methods

### Study design and population

This prospective, observational, multicenter cohort study included all STEMI patients diagnosed and treated within the period of December 1, 2013 to April 30, 2020 in the city of Cologne, Germany. The concept of the Cologne Infarction Model network (“Kölner Infarkt Modell”, KIM) has been described previously^14,15^. Briefly, KIM is a co-operation between all sixteen hospitals and the emergency medical services in the 400 km^2^ area of Cologne, Germany, populated by about 1 million people. Seven out of sixteen hospitals feature percutaneous coronary intervention (PCI) capabilities 24 h a day, 7 days a week. STEMI patients who first presented to emergency medical service were directly transferred to the catheterization laboratories of those PCI centers. Patients who presented to non-PCI hospitals were immediately transferred to hospitals with catheterization laboratory capabilities. Guideline conform STEMI diagnosis required typical symptoms in the presence of either ST-segment elevation in at least two contiguous leads or assumed new onset left bundle branch block (LBBB) on a 12-lead electrocardiogram^8,16^. All patients with STEMI and a complete report on treatment periods reflecting pre-clinical and in-hospital delay were eligible for the current analysis. The study complies with the Declaration of Helsinki. Ethical approval was issued by the local ethics committee of the University of Cologne (No. 06-064) and written informed consent was obtained from all patients. The KIM registry is funded by the KIM registered association.

### Observational period

The first wave of the COVID-19 pandemic began on January 27, 2020 and ended around April 30, 2020 in Germany^17^. We chose this timeframe for the COVID-19 pandemic observational group. This group was compared to a seasonal control group (pandemic season control group), including all patients treated between January 27 and April, 30 between the years 2014 to 2019. All other patients were excluded because of a potential seasonal effect on health care provision and patients’ behavior.

The Influenza 2017/2018 epidemic began on December 25, 2017 and ended around April 30, 2018 in Germany^10^. The Influenza 2017/2018 epidemic group itself was compared to a corresponding control group (epidemic season control group) treated between December 25 and April 30 during the prior years. Moreover, a direct comparison of the COVID-19 pandemic and the Influenza 2017/2018 epidemic group was performed.

### Treatment periods

To address the hypothesized effect of the COVID-19 pandemic and Influenza 2017/2018 epidemic on STEMI treatment time indicators were defined as follows: (1) symptom-to-contact time (S2C, defined as the period from symptom-onset to first medial contact), (2) contact-to-balloon time (C2B, defined as the period from first medical contact to balloon inflation), and (3) door-to-balloon time (D2B, defined as the period from arrival at the PCI-hospital to balloon inflation). Additionally, data on mortality, procedural results and peri-interventional complications were extracted from the registry. Peri-interventional data included the access route, culprit lesion, antiplatelet therapy, the maximum amount of creatin kinase, local bleeding rates and peri-interventional ventricular arrhythmia.

### Statistical analysis

Data were described using median [interquartile range], or mean values (± standard deviation), or frequencies and percentages. Extreme values suspected to be implausible data were censored. The Student’s t-test, Fisher’s exact test and Chi square test were used for statistical analyses. All reported *p*-values were two-sided, and *p*-values less than 0.05 were considered statistically significant. Statistical analyses were performed using SPSS Statistics Version 26.0.0 (NY: IBM Corp., Armonk).

### Ethics approval

The study complies with the Declaration of Helsinki. Ethical approval was issued by the local ethics committee of the University of Cologne.

### Consent to participate

Written informed consent was obtained from all patients.

## Results

1455 patients were eligible for statistical analysis. The COVID-19 pandemic group included 82 patients and the corresponding pandemic season control group had 358 patients. The Influenza 2017/2018 epidemic group included 54 patients, and the corresponding epidemic season control group consisted of 366 patients. The daily incidence of STEMI was 1.49 during the COVID-19 pandemic and 1.40 (*p* = 0.677) for the pandemic season control group. Corresponding daily STEMI incidence was 1.22 during the Influenza 2017/2018 epidemic, and 1.28 during the epidemic season control period.

### Pre-clinical and baseline data

Baseline characteristics are summarized in Table 1a, b. Mean age was 65.7 years in the COVID-19 pandemic group and 63.5 years in the Influenza 2017/2018 epidemic group. Corresponding mean age was 63.6 for seasonal epidemic and pandemic control group. Patients exhibited similar baseline characteristics regarding gender, heart rate at presentation, and initial systolic blood pressure. Documented ST-segment elevation in at least two contiguous leads during 12-lead ECG was the predominant manifestation of STEMI, whereas assumed new onset LBBB occurred less frequently. During the pre-clinical course, 18.2% of patients in the COVID-19 pandemic group suffered from cardiac arrest requiring resuscitation. The rate of cardiac arrest was numerically lower during the Influenza 2017/2018 epidemic (*p* = 0.299). None of the patients in the COVID-19 pandemic group had a SARS-CoV-2 infection. The results of influenza testing and the use of antiviral therapy were not documented in the KIM registry.

**Table 1:** Baseline characteristics.

	Total cohort N = 1455 (%)	COVID-19 pandemic group N = 82 (%)	Pandemic season control group N = 358 (%)	*p*-value		
**(a) Baseline characteristics COVID-19 pandemic analysis**						
Age, years, mean	63.4	65.7	63.6	0.192		
Gender, male	1010/1412 (71.7)	59/81 (72.8)	231/346 (66.7)	0.047		
Initial heart rate, bpm, mean	80	82	78.2	0.495		
Initial systolic blood pressure, mmHg, mean	139.5	132.4	138.1	0.593		
Preclinical cardiac arrest	181/1053 (17.2)	6/33 (18.2)	49/256 (19.1)	1.0		
*STEMI*						
New LBBB	55/1269 (4.3)	0/72 (0)	15/307 (4.9)	0.085		
ST-Segment elevation	1214/1269 (95.7)	72/72 (100)	292/307 (95.1)			

	Total cohort N = 1455 (%)	Influenza 2017/2018 epidemic group N = 54 (%)	Epidemic season control group N = 366 (%)	*p*-value	COVID-19 pandemic group N = 82 (%)	*p*-value (*Influenza epidemic vs. COVID-19 pandemic group*)
**(b) Baseline characteristics Influenza 2017/2018 epidemic analysis**						
Age, mean	63.4	63.5	63.6	0.891	65.7	0.571
Gender, male	1010/1412 (71.7)	41/54 (75.9)	231/352 (65.6)	0.152	59/81 (72.8)	0.841
Initial heart rate, bpm, mean	80	83	78	0.297	82	0.918
Initial systolic blood pressure, mmHg, mean	139.5	146.8	138.4	0.271	132.4	0.208
Preclinical cardiac arrest	181/1053 (17.2)	3/35 (8.6)	53/272 (19.5)	0.161	6/33 (18.2)	0.299
*STEMI*						
New LBBB	55/1269 (4.3)	1/51 (2)	14/382 (4.5)	0.705	0/72 (0)	0.415
ST-Segment elevation	1214/1269 (95.7)	50/51 (98)	296/310 (95.5)		72/72 (100)	

*STEMI* ST-segment elevation myocardial infarction, *LBBB* left bundle branch block.

### Treatment periods

#### COVID-19 pandemic

**Figure 1:**
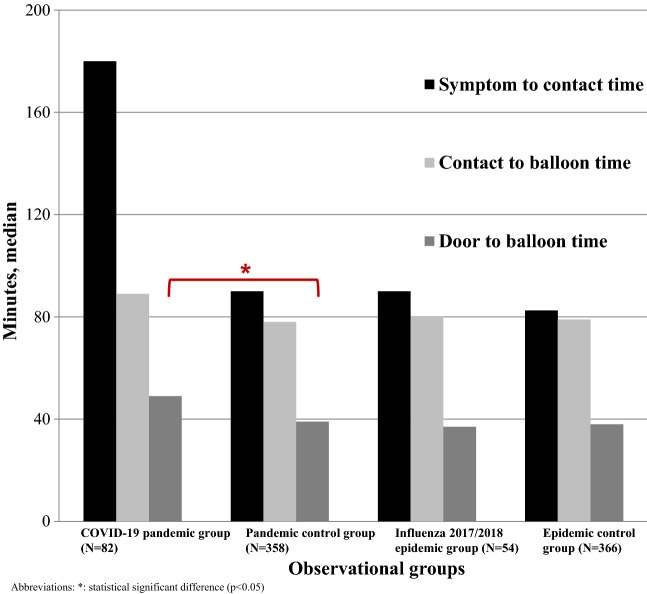
Treatment delay until revascularization.

The treatment periods are summarized in Table 2a, b, and visualized in Fig. 1. Patients in the COVID-19 pandemic group had a median S2C time of 180 [30–390] minutes which was longer than the pandemic season control group (90 [30–351] minutes, *p* = 0.183). The median C2B time was 89 [69–108] minutes for the COVID-19 pandemic and 78 [64–95] minutes for the pandemic season control group (*p* = 0.097). Corresponding times for the D2B interval were 49 [27.3–73.3] and 39 [29.8–51.3] minutes (*p* = 0.038). The median length of hospital stay was 5 days for both groups (*p* = 0.529).

**Table 2:** Time intervals.

	Total cohort N = 1455	COVID-19 pandemic group N = 82	Pandemic season control group N = 358	*p*-value		
**(a) Treatment periods COVID-19 pandemic analysis**						
S2C time	70 [30–246]	180 [30–390]	90 [30–352]	0.183		
C2B time	78 [64–94]	89 [69–108]	78 [64–95]	0.097		
D2B time	39 [29–55]	49 [27.3–73.3]	39 [29.8–51.3]	0.038*		
Length of hospital stay (days)	5 [4–7.5]	5 [4–7]	5 [3–7]	0.529		

	Total cohort N = 1455	Influenza 2017/2018 epidemic group N = 54	Epidemic season control group N = 366	*p*-value	COVID-19 pandemic group N = 82	*p*-value (*Influenza epidemic vs. COVID-19 pandemic group*)
**(b) Treatment periods Influenza 2017/2018 epidemic analysis**						
S2C time	70 [30–246]	90 [30–240]	82.5 [30–300]	0.788	180 [30–390]	0.216
C2B time	78 [64–94]	80 [63–97.5]	79 [63–95]	0.733	89 [69–108]	0.274
D2B time	39 [29–55]	37 [29.5–60]	38 [27–51]	0.429	49 [27.3–73.3]	0.421
Length of hospital stay (days)	5 [4–7.5]	5 [3–8.3]	5 [4–8]	0.816	5 [4–7]	0.834

*S2C* symptom-to-contact time, *C2B* contact-to-balloon time, *D2B* door-to-balloon time, *N2B* needle-to-balloon time; *: statistical significant difference.

Median [interquartile range].

#### Influenza 2017/2018 epidemic

Patients in the Influenza 2017/2018 epidemic group had a median S2C time of 90 [30–240] minutes, which was longer than the epidemic season control group (82.5 [30–300] minutes, *p* = 0.788). The median C2B time was 80 [63–97.5] minutes for the Influenza 2017/2018 epidemic and 79 [63–95] minutes for the epidemic season control group (*p* = 0.733). Corresponding D2B intervals were 37 [29.5–60] and 38 [27–51] minutes (*p* = 0.429). The median length of hospital stay was 5 days for both groups (*p* = 0.816).

In the comparison of both respiratory infectious diseases, the COVID-19 pandemic had longer S2C, C2B and D2B intervals, but none of these differences was significantly different (see Table 2b).

### Procedural data

The radial artery was the predominant access route in the COVID-19 pandemic and the Influenza 2017/2018 epidemic group (see Table 3). These proportions were statistically significant different compared to those from the seasonal control groups, as the femoral artery puncture were performed in 64.2 and 70.2 of all procedures in the historical cohorts (*p* < 0.001). Angiography revealed that LAD occlusion was the most frequent cause of STEMI in all patients, but the proportion of LAD occlusion was significantly higher in patients of the Influenza 2017/2018 epidemic group (55.6%) in comparison to the epidemic season control group (36.7%, *p* = 0.011). Myocardial infarction of the inferior wall either caused by occlusion of RCA or subsequent branches was the second most common cause for STEMI. After primary angiography 39 patients were transferred to urgent CABG operation. In the remaining cohort, interventional reperfusion (TIMI I-III) was achieved in all but 142 patients.

**Table 3:** Periprocedural characteristics.

	Total cohort N = 1455 (%)	COVID-19 pandemic group N = 82 (%)	Pandemic season control group N = 358 (%)	*p*-value (*COVID-19 pandemic vs. pandemic season control group*)	Influenza 2017/2018 epidemic group N = 54 (%)	Epidemic season control group N = 366 (%)	*p*-value (*Influenza 2017/2018 epidemic vs. epidemic season control group*)	*p*-value (*Influenza 2017/2018 epidemic vs. COVID-19 pandemic group*)
**Access route**								
Radial access	540/1303 (41.4)	55/82 (67.1)	122/341 (35.8)	< 0.001*	30/47 (63.8)	104/349 (29.8)	< 0.001*	0.705
Femoral access	763/1303 (58.6)	27/82 (32.9)	219/341 (64.2)		17/47 (36.2)	245/349 (70.2)		
**Culprit lesion**^**a**^								
LAD	585/1404 (41.7)	41/82 (50.0)	140/357 (39.2)	0.082	30/54 (55.6)	134/365 (36.7)	0.011*	0.600
RD	47/1404 (3.3)	4/82 (4.9)	10/357 (2.8)	0.308	1/54 (1.9)	12/365 (3.3)	1.0	0.648
CFX	195/1404 (13.9)	6/82 (7.3)	57/357 (16.0)	0.053	8/54 (14.8)	58/365 (15.9)	1.0	0.248
RPLS	69/1404 (4.9)	2/82 (2.4)	22/357 (6.2)	0.280	1/54 (1.9)	29/365 (7.9)	0.154	1.0
RCA	459/1404 (32.7)	32/82 (39.0)	117/357 (32.8)	0.302	14/54 (25.9)	122/365 (33.4)	0.350	0.140
LIMA-LAD Bypass graft	5/1404 (0.4)	1/82 (1.2)	1/357 (0.3)	0.339	0/54 (0)	2/365 (0.5)	1.0	1.0
Venous Bypass graft	16/1404 (1.1)	2/82 (2.4)	3/357 (0.8)	0.235	0/54 (0)	3/365 (0.8)	1.0	0.518
Other	36/1404 (2.6)	3/82 (3.7)	11/357 (3.1)	0.732	0/54 (0)	10/365 (2.7)	0.373	0.276
**Postprocedural antiplatelet therapy**								
ASA + Clo	196/1261 (15.5)	8/75 (10.7)	53/322 (16.5)	0.285	6/48 (12.5)	61/339 (18.0)	0.419	0.777
ASA + Pra	461/1261 (36.6)	32/75 (42.7)	114/321 (35.4)	0.288	11/48 (22.9)	140/338 (41.4)	0.017*	0.033*
ASA + Tic	433/1261 (34.3)	29/75 (38.7)	110/322 (34.2)	0.502	24/48 (50.0)	91/339 (26.8)	0.002*	0.264
Other	171/1261 (13.6)	6/75 (8.0)	45/322 (14.0)	–	7/48 (14.6)	47/339 (13.9)	–	–
**Maximum of CK**								
Mean, U/L	2115.42	2058.86	2147.98	0.214	2061.86	2081.8	0.988	0.338

*LAD* left artery descending, *RD* diagonal branch, *CFX* circumflex artery, *RPLS* posterolateral artery branch, *RCA* right coronary artery, *LIMA* left internal mammary artery, *ASA* acetylsalicylic acid, *Clo* Clopidogrel, *Pra* Prasugrel, *Tic* Ticagrelor, *CK* creatin kinase, *: statistical significant difference.

^a^In case of multiple occlusions multiple vessels were considered.

Peri-interventional complications occurred in 8.5% of patients in the COVID-19 pandemic and 5.9% of patients in the Influenza 2017/2018 epidemic group (see Table 4). The corresponding rates were 14.6% and 14.8% for the seasonal control groups. Specifically, in-hospital mortality was 6.1% for COVID-19 pandemic group and 11.0% for the pandemic season control group (*p* = 0.129). Corresponding rates were 5.9% for the Influenza 2017/2018 epidemic and 11.2% for the related epidemic season control group (*p* = 0.333).

**Table 4:** Periprocedural complication rates.

	Total cohort N = 1455 (%)	COVID-19 pandemic group N = 82 (%)	Pandemic season control group N = 358 (%)	*p*-value (*COVID-19 pandemic vs. pandemic season control group*)	Influenza 2017/2018 epidemic group N = 54 (%)	Epidemic season control group N = 366 (%)	*p*-value (*Influenza 2017/2018 epidemic vs. epidemic season control group*)	*p*-value (*Influenza 2017/2018 epidemic vs. COVID-19 pandemic group*)
Death	146/1398 (10.4)	5/82 (6.1)	39/355 (11.0)	0.225	3/51 (5.9)	41/366 (11.2)	0.333	1.0
Re-Infarction	12/1345 (0.9)	2/82 (2.4)	5/341 (1.5)	0.625	0/50 (0)	6/350 (1.7)	1.0	0.526
Ventricular fibrillation	22/1345 (1.6)	0/82 (0)	6/341 (1.8)	0.602	0/50 (0)	3/349 (0.9)	1.0	–
Access route bleeding	11/1345 (0.8)	0/82 (0)	2/341 (0.6)	1.0	0/50 (0)	4/349 (1.1)	1.0	–

*LAD* left artery descending, *RD* diagonal branch, *CFX* circumflex artery, *RPLS* posterolateral artery branch, *RCA* right coronary artery, *LIMA* left internal mammary artery, *ASA* acetylsalicylic acid, *Clo* Clopidogrel, *Pra* Prasugrel, *Tic* Ticagrelor, *CK* creatin kinase, *: statistical significant difference; –: Testing not applicable.

## Discussion

### Major findings

The incidence of STEMI in the metropolitan area of Cologne was steady during the first wave of COVID-19 pandemic or the Influenza 2017/2018 epidemic. We observed adequate intervals between first medical contact and revascularization. In detailed analysis, both diseases demonstrated a trend towards an increased patient-related delay compared to each corresponding seasonal control group without significant differences. Additionally, the current analysis showed a prolonged door-to-balloon time during the COVID-19 pandemic. The direct comparison of the COVID-19 pandemic and Influenza 2017/2018 epidemic did not demonstrate a significant difference regarding system- or patient-related delay.

### STEMI incidence

The incidence of STEMI was not significantly affected by the respiratory virus diseases in this registry analysis. To our knowledge, this is the first analysis on the effect of the Influenza 2017/2018 season on STEMI care. Over the past years, only few studies investigating the effect of influenza season on myocardial infarction and especially on STEMI incidence or care were published^11–13^. An analysis of the SWEDEHEART registry demonstrated an increased risk of myocardial infarction during the yearly influenza season and a correlation with influenza burden^11^. In a subgroup analysis STEMI patients were not at higher risk for cardiovascular mortality during the influenza season^11^. This result is in line with the current study^11^. Registry data in the United States demonstrated that influenza infection was an independent predictor of in-hospital mortality in patients with myocardial infarction^13^. Concomitant influenza infection and myocardial infarction reduced the rate of revascularization rate significantly^12,13^.

On the contrary, a large amount of studies regarding the COVID-19 pandemic and the effect on patients with STEMI have been published over the last year. Studies done in Europe and China documented a decrease in hospital admissions for STEMI^2,18–20^. De Rosa et al. reported a 26.5% reduction of STEMI admission during a 1 week period and Scholz et al. detected a 12.6% decrease during a 1 month period^18,20^. All of these studies were observational studies or registries and might have underreported the true incidence of STEMI during the respiratory virus epidemic and pandemic.

### Treatment delay

Regarding the STEMI treatment during the COVID-19 pandemic the S2C, C2B and D2B were longer compared to the pandemic season control group, but only the D2B was significantly prolonged. The median S2C was 180 min and this was a doubling in comparison to the pandemic season control group. As this was not a statistical significant difference, careful interpretation is required. Nevertheless this difference might have clinical impact and is in line with observations in Europe and Asia. De Rosa et al. and Gramegna et al. observed a relevant patient-related delay with a prolonged symptom-to-hospital and contact-to-balloon time during the COVID-19 pandemic^18,21^. Interestingly, these studies took place in outbreak areas during the climax of the early COVID-19 pandemic in Italy. A German analysis of 15,800 patients documented comparable S2C, C2B and D2B times for the COVID-19 pandemic and control group without any significant differences^20^. Multiple studies documented an increase or a trend towards a patient-related delay during the COVID-19 pandemic, but the extent differs between the trials^3–5,19,20^. One potential reason for divergent results is the variance in time frames of COVID-19 pandemic groups. Scholz et al. and Xiang et al. investigated a 1-month period, whereas De Rosa chose a 1-week observation period^18–20^. Daoulah et al. extended the investigation period by analyzing all patients treated over a 4 month period between January 1, 2020 and April 30, 2020^5^. As a result of these heterogeneous approaches, the aforementioned studies came to inconsistent conclusions about the effect of the COVID-19 pandemic on STEMI patient care.

The reasons for patient-related delay are still under consideration. Altruistic behavior has been intensively discussed. One might further argue that patients avoided hospitals as they represent the focus of this viral disease, and because hospitals were confronted by an excessive demand of intensive care unit capacities by COVID-19 patients. These circumstances might also be an explanation for the observed prolonged D2B time in the current analysis. Results by a meta-analysis from Rattka et al. evolving around 50,000 participants also demonstrated a similar increase in D2B time for the COVID-19 group^22^.

### Mortality

The registry data indicate a deceased mortality during the Influenza 2017/2018 epidemic and COVID-19 pandemic period. In the setting of the COVID-19 pandemic, we speculate that the availability of resources due to the reduction in elective procedures could have had an impact on these outcomes. On the other hand, a potentially increased local sudden cardiac death rate might have improved the in-hospital outcome (survivorship bias). The current local death rate of the city of Cologne, Germany is not available, hence the true explanation of this phenomenon is still pending and might be addressed in future studies.

### Limitations

Cologne was not highly affected during the observational period of the COVID-19 pandemic^17^. Until April 30, 2020, less than 0.24% (N = 2346) of the total population had a SARS-CoV-2 infection^17^. In contrast, reports by De Rosa et al. and Gramegna et al. included hospitals from outbreak areas with much higher incidence of COVID-19 infections. This might not only have influenced the system, but also the patient-related delay^18,21^. Inherent limitations of registry studies also affected the current analysis. A short follow-up period, underreporting of outcome data, limited availability of patient characteristics including comorbidities and treatment data leading to missing data restrict the generalizability of our results. Assessment of subjective variables (e.g. symptom onset) and data management by diverse centers might also influence the study results. One additional limitation is the lack of information on the incidence of influenza infection or antiviral treatment in the cohorts. Unfortunately these variables are not part of the KIM registry, but might have biased the current analysis.

Another important aspect is the local structure of Cologne with a dense structure of hospitals with short transfer periods and the small sample size in the KIM registry. The current analysis is also restricted by the low number of events especially in the observational groups.

## Conclusion

A well-structured co-operation of emergency medical service and local hospitals in a metropolitan area was able to overcome the challenges in the treatment of patients with STEMI during the COVID-19 pandemic and the Influenza 2017/2018 epidemic. In the setting of this network, these respiratory infectious diseases did not result in a significant overall treatment delay. These virus infections did neither cause an increase in “STEMI incidence” section, nor did they raise the STEMI mortality. Nonetheless, both diseases showed a trend towards an increased patient-related delay. Educational programs should address the observed patient-related delay and emphasize the need for immediate contact of the medical system. Additionally, the results demonstrated a significantly prolonged door-to-balloon time during the COVID-19 pandemic. Adequate strategies to minimize this delay and improve health care service balancing prompt treatment and infection protection are needed.

## Abbreviations

ASA: Aspirin, acetylsalicylic acid
CABG: Coronary artery bypass graft
CK: Creatine kinase
CFX: Circumflex artery, Ramus circumflexus
Clo: Clopidogrel
ECG: Electrocardiogram
EMS: Emergency medical service
KIM: The Cologne Infarction Model Registry
LAD: Left artery descending
LBBB: Left bundle branch block
LIMA: Left internal mammary artery
PCI: Percutaneous coronary intervention
Pra: Prasugrel
RCA: Right coronary artery
RD: Diagonal branch, Ramus diagonalis
RPLS: Posterolateral artery branch, Ramus posterolateralis
SARS-CoV-2: Severe acute respiratory syndrome coronavirus 2
STEMI: ST-elevation myocardial infarction
Tic: Ticagrelor
TIMI flow grade: Thrombolysis in myocardial infarction flow grade
